# Temporal Trend of the Prevalence of Modifiable Risk Factors of Stroke: An Ecological Study of Brazilians between 2006 and 2012

**DOI:** 10.3390/ijerph19095651

**Published:** 2022-05-06

**Authors:** Laércio da Silva Paiva, Luiz Vinicius de Alcantara Sousa, Fernando Rocha Oliveira, Luis Eduardo Werneck de Carvalho, Rodrigo Daminello Raimundo, João Antonio Correa, Luiz Carlos de Abreu, Fernando Adami

**Affiliations:** 1 Laboratório de Epidemiologia e Análise de Dados, Departamento de Saúde da Coletividade, Centro Universitário FMABC, Santo André 09060-870, Brazil; luiz.sousa@fmabc.br (L.V.d.A.S.); fernando.adami@fmabc.br (F.A.); 2 Departamento de Epidemiologia, Faculdade de Saúde Pública, Universidade de São Paulo, São Paulo 01246-904, Brazil; oliveira.fernando.rocha@hotmail.com; 3 Oncológica do Brasil Ensino e Pesquisa, Belém 66053-350, Brazil; dreduardocarvalho@oncologicadobrasil.com.br; 4 Laboratório de Delineamento de Estudos e Escrita Científica, Departamento de Saúde da Coletividade, Centro Universitário FMABC, Santo André 09060-870, Brazil; rodrigo.raimundo@fmabc.br; 5 Disciplina de Angiologia e Cirurgia Vascular, Centro Universitário FMABC, Santo André 09060-870, Brazil; cor.jantonio@gmail.com; 6 Departamento de Saúde Integrada em Saúde, Universidade Federal do Espirito Santo—UFES, Vitoria 29075-910, Brazil; luiz.abreu@ufes.br

**Keywords:** stroke, epidemiology, risk factors, prevalence

## Abstract

Stroke is one of the leading causes of death worldwide, including in Brazil. This study aimed to analyze the temporal trend of the prevalence of modifiable risk factors of stroke from 2006 to 2012. This ecological study was conducted by secondary analysis in May 2018, using data from the surveillance of risk factors and protection for chronic diseases by telephone inquiry (VIGITEL) available in the Department of Informatics of the Unified Health System (DATASUS). The modifiable risk factors of stroke in Brazilians were systemic arterial hypertension, diabetes mellitus, abusive alcohol consumption, overweight, and obesity. Overall, there was a significant increase in the risk factors of diabetes (β = 0.30, *P* = 0.001, r^2^ = 0.99), overweight (β = 0.50, *P* = 0.002, r^2^ = 0.98), and obesity (β = 0.88, *P* < 0.001, r^2^ = 0.96). However, there was a stability in the prevalence of hypertension (β = 0.25, *P* = 0.320, r^2^ = 0.88) and alcohol abuse (β = 0.32, *P* = 0.116, r^2^ = 0.49). There was an increase in the prevalence of diabetes mellitus, overweight, and obesity, but stability in systemic arterial hypertension and abusive alcohol consumption in the Brazilian population.

## 1. Introduction

Stroke is one of the leading causes of death worldwide, including in Brazil [1]. In the United States, stroke is the fourth most common cause of death, the second most common cause of dementia, and the most common cause of disability among adults [2]. Among the causes of mortality in Brazil, cerebrovascular diseases closely follow acute myocardial infarction and pneumonia [3].

Previous research has reported a reduction in the stroke-related mortality rate in the world of 36.0% (31.0–42.0) between 1990 and 2019 [4], which has been observed in Brazil, 50.4% in men and 49.6% in women [5], and in different regions of Brazil between 1997 and 2012 [6]. In addition, the overall stroke incidence significantly increased by 62% (incidence rate ratio, 1.62; 95% confidence interval [CI], 1.10–2.40) [7]. In Brazil, the incidence of hospital admissions for stroke was 24.67% (CI 95% 24.66; 24.67) in 2008 and 25.11% (95% CI 25.10; 25.11) in 2012 [4]. However, the number of risk factors of stroke has also increased [8].

In general, the predisposing risk factors of stroke are classified as non-modifiable and modifiable [2]. Non-modifiable risk factors include sex, age, ethnicity, family history, and geographic location, which serve as markers for high-risk stroke. Modifiable risk factors include diabetes mellitus, systemic hypertension, hyperlipidemia, atrial fibrillation, coronary artery disease, obesity, and smoking, which are amenable to intervention and present a lower risk for stroke. Additionally, newer risk factors include hyperhomocysteinemia, hypercoagulability states, and certain biomarkers [2,9]. Globally, more than 90% of the stroke burden is attributed to modifiable risk factors [10]. A study by Kotlega et al. [11] showed that the pre-hospital detection of risk factors of stroke, such as hypertension, coronary heart disease, atrial fibrillation, diabetes, and dyslipidemia, increased significantly after 10 years. However, atrial fibrillation was the single most frequently detected risk factor pre- and intra-hospital after 10 years. However, a study proposed by Soto-Câmara et al. [12] showed that knowledge of warning signs or risk factors of stroke is low in hospitalized patients, with only a higher education level (odds ratio [OR] = 3.19; 95% CI: 1.70; 5.74, *P* = 0.003) and history of the previous stroke (OR = 3.54; 95% CI: 2.09; 5.99; *P* < 0.001) being factors associated with a greater degree of knowledge of the warning signs of stroke.

Further consideration should be given depending on the presence and intensity of risk factors, the chances of developing stroke are greater, and sequelae are severe. Research indicates that the presence of type I and II diabetes mellitus is related to the risk of developing a stroke. There is also sufficient evidence to show the relationship between the number of cigarettes consumed per day and the risk of stroke. However, abusive alcohol consumption, its actual potential to harm the individual, and its relation to stroke, have been the subject of debate [13,14,15,16]. A recent study by Soto-Câmara et al. [17] showed that active smoking, sedentary lifestyles, heavy drinking, or being overweight or obese increased the risk of having an acute cerebrovascular event in individuals aged 75 years or younger.

Thus, in the analysis of data from the global literature, the question of the behavior of modifiable risk factors of stroke in Brazil arises. Our results could add to the identification of factors that influence the epidemiology of stroke, which is relevant to the appropriate policies and protocols and should remain the focus of public health activities [18]. Therefore, the present study aimed to analyze the temporal trend of the prevalence of modifiable risk factors of stroke among Brazilians between 2006 and 2012.

## 2. Method

An ecological study was conducted in May 2018 by secondary analysis of the prevalence data of modifiable risk factors of stroke, obtained from the surveillance of risk factors and protection for chronic diseases by telephone inquiry (VIGITEL) available on the website of the Department of Informatics of the Unified Health System (DATASUS). The DATASUS is a Brazilian health information database that is publicly available and free of charge through the link (http://www2.datasus.gov.br/ accessed on 31 May 2018) [19].

VIGITEL information was collected via telephone interviews conducted between 2006 and 2012 from an adult population (≥18 years old) living in households with at least one fixed telephone line in the 26 capitals of the Brazilian states and the Federal District [20]. Approximately 54,000 individuals responded to the survey each year using probabilistic sampling [21].

Risk factors such as diabetes, high blood pressure, abusive alcohol consumption, overweight, and obesity were self-reported during the telephone survey using questions in the questionnaire defined by the VIGITEL System (Table 1).

Data on risk indicators and protective factors for diabetes mellitus, systemic arterial hypertension, abusive alcohol consumption, and obesity in adults, according to the Brazilian indicators and basic data (2012), were collected. These were stratified by age groups (18–24, 25–34, 35–44, 45–54, 55–64, and ≥65 years), regions of Brazil (North, Northeast, Southeast, South, and Central West), and the period between 1 January 2006 and 31 December 2012, from the DATASUS database. Notably, there were no diabetes mellitus data for the 18–34 years age group.

Data collection in the DATASUS system was performed by two independent researchers using extraction worksheets made by the authors, and a third researcher was responsible for necessary corrections in the case of a data discrepancy. As this is public data of national scope in Brazil, there was no need for approval by the research ethics committee, according to the Resolution of the National Health Council (CNS) No. 466 of 12 December 2012.

The prevalence rates of risk factors of stroke were calculated using the following formulas [19]: 

Diabetes mellitus ^#^

$$\frac{number\;of\;adults\;\left(\geq 35\;years\;of\;age\right)\;with\;diabetes}{\mathrm{number}\;\mathrm{of}\;\mathrm{interviewed}\;\mathrm{adults}\;\mathrm{aged}\;\geq 35\;\mathrm{years})}\times 100$$

Systemic arterial hypertension ^#^:

$$\frac{number\;of\;adults\;\left(\geq 18\;years\;of\;age\right)\;with\;referred\;systemic\;hypertension}{\mathrm{number}\;\mathrm{of}\;\mathrm{interviewed}\;\mathrm{adults}\;\mathrm{aged}\;\geq 18\;\mathrm{years}\;}\times 100$$

Abusive alcohol consumption ^#^:

$$\frac{number\;of\;adults\;\left(\geq 18\;years\;of\;age\right)\;with\;abusive\;alcoholic\;beverage}{\mathrm{number}\;\mathrm{of}\;\mathrm{interviewed}\;\mathrm{adults}\;\mathrm{aged}\;\geq 18\;\mathrm{years}\;}\times 100$$

Overweight and obesity:

$$\frac{number\;of\;individuals\;\geq 18\;years\;of\;age\;with\;BMI\;\left(reference\;*\right)}{\mathrm{number}\;\mathrm{of}\;\mathrm{interviewed}\;\mathrm{adults}\;\mathrm{aged}\;\geq 18\;\mathrm{years}\;}\;\times \;100$$

* Overweight (BMI ≥ 25.0 and < 30.0 kg/m^2^) and obesity (BMI ≥ 30.0 kg/m^2^) ^#^ Percentage weighted to adjust the sociodemographic distribution of the VIGITEL sample to the distribution of the adult population of the city in the demographic census of 2000.

Prevalence estimates and confidence intervals (95% CI) were calculated by sex, age, and education level. Data analysis considered weighting factors that included the greater probability that individuals with more telephone numbers or fewer residents in the household participated in the sample, in addition to correcting the overestimation or underestimation of the VIGITEL sample resulting from telephone coverage unequal fixed in the studied locations, according to the sociodemographic strata and the sample size of each city. A detailed description of the methodological aspects of VIGITEL can be found in Brasil (2013) [22].

### Statistical Analysis

The prevalence rate was used to describe the epidemiological data. Prais–Winsten regression analysis was used to determine the temporal behavior of risk factors of stroke between 2006 and 2012, stratified by age group and geographic regions of Brazil. For the analysis of temporal trends, the methodological indications proposed by Antunes et al. were used [23]. The construction of the time series was calculated using Prais–Winsten regression, which allowed first-order autocorrelation corrections to be performed on values organized by time. Thus, the following data were estimated: the angular coefficient (β), probability value (p), and predictive capacity (r^2^) of the model.

Additionally, the Durbin–Watson test was used [23], and the growth or decline rates were calculated according to the angular coefficient values, specific by age group, Brazil, and regions. This procedure facilitated the classification of time trend rates as increasing, decreasing, or stationary.

The confidence level adopted for all analyses was 95%, and the statistical program used was Stata (Data Analysis and Statistical Software), version 11.0.

## 3. Results

The prevalence rates of modifiable risk factors of stroke in the Brazilian population between 2006 and 2012 were analyzed.

The prevalence of diabetes mellitus increased significantly in the 35–44 (β = 0.12, *P* = 0.003, r^2^ = 0.98) and ≥65 years age groups (β = 0.62, *P* = 0.021; r^2^ = 0.62) but remained stable in the 45–64 years age group. In the Brazilian regions, there was a significant increase in the prevalence of diabetes mellitus in all regions, except in the Northern region, with a higher prevalence in the Midwest region (β = 0.39, *P* < 0.001, r^2^ = 0.99) and a lower prevalence in the Northeast region (β = 0.23, *P* = 0.012, r^2^ = 0.77) (Table 2).

The analysis of the prevalence of systemic arterial hypertension in all age groups (≥18 years) showed stability in the prevalence rate throughout the analyzed period, with the highest rates in the oldest age group (≥65 years). In the Brazilian regions, there was a significant increase in the Central West (β = 0.56, *P* = 0.037, r^2^ = 0.76) and South (β = 0.43, *P* = 0.014, r^2^ = 0.97), whereas the other regions showed stability during this period (Table 3).

When comparing the rate of abusive alcohol consumption among adults, a significant increase was seen in the 55–64 (β = 0.63, *P* = 0.008, r^2^ = 0.74) and ≥65 years age groups (β = 0, 43; *P* < 0.001; r^2^ = 0.94). Among the Brazilian regions, only the Southeast region showed a significant increase (β = 0.47, *P* = 0.033, r^2^ = 0.75) (Table 4).

Regarding the overweight rate, there was a significant increase in the 18–24 (β = 0.85, *P* = 0.0061, r^2^ = 0.90), 25–34 (β = 0.77, *P* < 0.001; r^2^ = 0.99), and ≥65 years age groups (β = 0.35, *P* = 0.011, r^2^ = 0.99). Among the Brazilian regions, all regions showed a significant increase, except in the Southeast region. The highest increase over time was found in the Midwest (β = 0.82, *P* < 0.001, r^2^ = 0.99), Northeast (β = 0.70, *P* < 0.001, r^2^ = 0.83), and North (β = 0.68, *P* = 0.001; r^2^ = 0.83) regions (Table 5).

A significant increase in the prevalence rate was seen in all age groups with respect to obesity. The 35–44 years age group (β = 1.13, *P* < 0.001; r^2^ = 0.93) showed a greater increase over time. All Brazilian regions showed a significant increase, with the highest increase reported in the North region (β = 0.86, *P* < 0.001; r^2^ = 0.98) (Table 5).

**Figure 1:**
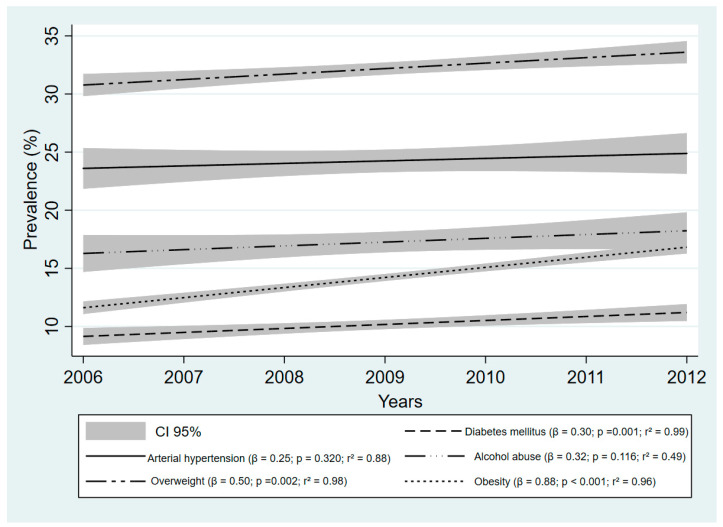
Prevalence of major modifiable risk factors of stroke among Brazilian adults between 2006 and 2012. **Data Source:** Surveillance of Risk Factors and Protection for Chronic Diseases by Telephone Inquiry (VIGITEL).

When assessing the modifiable risk factors of stroke among the Brazilian population from 2006 to 2012, all risk factors demonstrated an increase. However, there was a significant increase in the prevalence of risk factors of diabetes (β = 0.30, *P* = 0.001; = 0.99), overweight (β = 0.50, *P* = 0.002, r^2^ = 0.98), and obesity (β = 0.88, *P* < 0.001, r^2^ = 0.96) (Figure 1).

## 4. Discussion

This study analyzed the time trends of the prevalence of modifiable risk factors (i.e., diabetes mellitus, hypertension, abusive alcohol consumption, overweight, and obesity) for stroke in Brazilians between 2006 and 2012. The participants were stratified by age group and country for analysis. It was found that the prevalence of diabetes, overweight, and obesity increased in the 6-year period, with no decrease in the prevalence of any risk factors.

The prevalence of hypertension remained stable in all age groups but increased in the Central West and Southern regions. Diabetes increased in the 35–44 and ≥65 years age groups and all regions, except in the North region. Otite et al. [24] reported approximately 1 million cases of stroke in the period 2004–2014 in the USA. Most patients (92.5%) had at least one risk factor, and this ratio increased in almost 6% of the patients over 10 years. Comorbidities were common among patients and increased during the 10-year period [24]. Various comorbidities, including hypertension and diabetes, enhance white matter injury and inhibit repair [25]. The prevalence of hypertension and diabetes is approximately 80% and 34%, respectively, in patients who have undergone a stroke [24]. Although the prevalence of hypertension in the Brazilian population decreased by approximately 6% between 1980 and 2010, it was still high (approximately 30%) [26]. Despite our results showing stability in hypertension prevalence, it is a common risk factor among patients who have undergone a stroke [25,27]. Diabetes prevalence could be higher in patients with hypertension than in the general population, which is probably due to endothelial injuries caused by diabetes that contribute to the development of hypertension [28].

The aggregation of risk factors suggests an increased risk of stroke. A higher BMI (i.e., being overweight and obese) has been associated with an increased prevalence of hypertension in the adult Brazilian population [28]. Obesity prevalence increased in all age groups and in all regions. The imbalance between energy intake and expenditure is the main cause of overweight and obesity, which has shown an increased prevalence worldwide [29]. We showed that abusive alcohol consumption increased in both the > 55 years age group and Southeast region, which is the most developed region in Brazil [30]. According to the World Health Organization, the world’s highest alcohol consumption levels are found in developed countries, indicating that high-income countries generally have the highest alcohol consumption [31]. Risk factors can be associated with each other. For example, abusive alcohol consumption might contribute to excessive energy consumption, which is associated with weight gain [32], and obesity is the main risk factor of hypertension and diabetes [29].

In the ≥65 years age group, all the risk factors increased, except for systemic arterial hypertension; thus, it can be concluded that aging is a risk factor of stroke [27,33,34]. If older individuals present with an increasing prevalence of risk factors, as we have shown, this population could be at a higher risk of stroke. The older adult population normally faces greater stroke severity [35], with impaired white matter recovery [25,27]. Furthermore, aging was inversely associated with knowledge of the risk factors of stroke among the German population [36]. In other age groups, we found an increased prevalence of risk factors. Li et al. [35] showed a decline in the age of stroke onset over a 13-year period. The prevalence of risk factors in the middle-aged groups could contribute to stroke at younger ages, which effectively means patients living with more years of stroke-related disability [35].

Nevertheless, the prevalence of classic cardiovascular disease risk factors remained largely unchanged in Augsburg, Southern Germany, a high-income region [18]. The authors suggest that this stability in the prevalence rates of risk factors remained in accordance with their findings of stable incidence rates of stroke [18]. We determined the prevalence of different risk factors in Brazil. The influence of socioeconomic and cultural factors on health determinants may explain this result. Brazil has great regional inequalities [30], where historical heritage demarcates territory use and the political and economic setup of the country [30].

Variations in the prevalence of risk factors associated with vascular diseases in the population according to the geographic region have been observed in several studies in European countries [37,38]. In Brazil, this differentiation can be attributed to population characteristics, geographic differences, lifestyle, and cultural habits, as mentioned above. However, there are no specific analyses or comparisons in the literature attributed to regional reasons and conditions for risk factors of stroke.

Feigin et al. [10] showed significant increases in low- and middle-income countries and significant decreases in high-income countries related to stroke-related disability-adjusted life-years, which were attributed to most modifiable risk factors between 1990 and 2013. Moreover, in this study, we found diets high in sodium, poor diets, and high tobacco smoking in low-income countries [10]. Socioeconomic factors could influence stroke prevalence [34] and mortality rates, as we have shown previously, with an inverse association between stroke mortality and human growth index, education, longevity, and income [39]. Müller-Nordhorn et al. [36] showed that 68% of participants were able to correctly identify more than one risk factor of stroke. The ability to name the four correct risk factors (maximum) was significantly associated with a higher educational level, a family history of stroke, and having received information about risk factors of stroke during the previous year. Moreover, the author showed that individuals with an increased stroke risk (i.e., older people or people from impaired socioeconomic backgrounds) had less knowledge of risk factors of stroke [36].

We showed both a stable and increased prevalence of risk factors of stroke over a 6-year period. None of the risk factors showed a decrease in the prevalence. These results emphasize the need for the detection and management of major risk factors such as hypertension, coronary heart disease, atrial fibrillation, diabetes, and dyslipidemia in primary and secondary prevention and knowledge of the risk factors of stroke in the general population [11].

The present study has certain limitations. We could not analyze important risk factors, such as tobacco use, dyslipidemia, cholesterol, and physical activity levels. Data on risk factors available in the DATASUS were only from 2006 to 2012. Data collected by telephone surveys of risk factors were self-reported, which may present some information bias. Another possible limitation of this study is the ecological fallacy, which makes it difficult to draw conclusions for individuals. Future investigations are needed to update the time trends of the risk factors.

## 5. Conclusions

Between 2006 and 2012, there was a significant increase in the prevalence of diabetes mellitus, overweight, and obesity; however, there was stability in the prevalence of systemic arterial hypertension and abusive alcohol consumption in the Brazilian population for the same period.

## Figures and Tables

**Table 1:** Description of the variables of the questionnaire proposed by VIGITEL to define Diabetes, Arterial Hypertension, Abusive consumption of alcohol, Overweight and Obesity.

Risk Factors	Variables
Diabetes	Positive answer to the question: “*Has a doctor ever told you that you have diabetes?*”.
Arterial Hypertension	Positive answer to the question: “*Has a doctor ever told you that you have high blood pressure?*”
Abusive consumption of alcohol	Abusive consumption of alcohol was considered to be five or more drinks for men or four or more drinks for women on a single occasion, at least once in the last 30 days. A dose of alcoholic beverage corresponds to a can of beer, a glass of wine or a dose of cachaça, whiskey or any other distilled alcoholic beverage.
Overweight	Individuals with a BMI ≥ 25 kg/m^2^, calculated from self-reported height and weight.
Obesity	Individuals with a BMI ≥ 30 kg/m^2^, calculated from self-reported height and weight.

**Table 2:** Prevalence of diabetes in adults reporting previous medical diagnosis, stratified by age group and regions of Brazil between 2006 and 2012.

Variables	Prevalence (95% CI)							Prais–Winsten Regression			
	2006	2007	2008	2009	2010	2011	2012	β	*P*	r^2^	Trend
**Age Group (years)**											
35 to 44	2.9 (2.3; 3.5)	2.9 (2.3; 3.4)	3.4 (2.7; 4.2)	3.3 (2.5; 4.0)	3.4 (2.7; 4.0)	3.3 (2.6; 4.1)	3.9 (3.0; 4.9)	0.12	0.003	0.98	Increase
45 to 54	7.1 (6.0; 8.2)	7.7 (6.6; 10.1)	9.0 (7.7; 10.4)	7.4 (6.3; 8.5)	8.1 (7.0; 9.2)	8.7 (7.6; 9.8)	9.3 (8.0; 10.6)	0.25	0.053	0.87	Stable
55 to 64	15.8 (13.7; 18.0)	15.6 (13.7; 17.6)	15.7 (13.8; 17.6)	15.3 (13.4; 17.2)	16.4 (14.6; 18.3)	14.8 (13.2; 16.5)	18.5 (16.6; 20.4)	0.06	0.118	0.49	Stable
≥65	19.2 (17.3; 21.1)	18.6 (16.6; 20.6)	21.2 (19.1; 23.3)	22.4 (20.2; 24.7)	21.9 (19.9; 23.8)	21.4 (19.5; 23.3)	22.9 (20.9; 25.0)	0.62	0.021	0.62	Increase
**Regions**											
North	8.8 (7.6; 9.9)	7.9 (6.9; 8.9)	7.4 (6.4; 8.3)	7.8 (6.9; 8.7)	8.7 (7.7; 9.7)	9.5 (8.5; 10.5)	8.4 (7.4; 9.3)	0.09	0.567	−0.48	Stable
Northeast	8.9 (8.1; 9.6)	9.5 (8.7; 10.3)	10.0 (9.1; 10.9)	10.1 (9.2; 10.9)	9.6 (8.8; 10.3)	10.3 (9.6; 11.1)	10.7 (9.9; 11.5)	0.23	0.012	0.77	Increase
Southeast	9.7 (8.5; 10.9)	9.7 (8.5; 10.9)	11.5 (10.2; 12.8)	10.8 (9.4; 12.1)	11.3 (10.1; 12.5)	10.7 (9.5; 11.9)	12.9 (11.4; 14.3)	0.35	0.008	0.98	Increase
South	8.5 (7.4; 9.6)	9.4 (8.2; 10.7)	9.0 (7.9; 10.1)	9.7 (8.6; 10.9)	10.8 (9.6; 12.0)	9.4 (8.4; 10.5)	12.5 (11.2; 13.8)	0.37	0.001	0.94	Increase
Midwest	8.3 (7.1; 9.5)	7.6 (6.6; 8.6)	9.2 (8.1; 10.4)	8.5 (7.3; 9.7)	9.7 (8.0; 11.3)	9.4 (8.4; 10.5)	10.5 (9.4; 11.6)	0.39	<0.001	0.99	Increase

**Data Source:** Surveillance of Risk Factors and Protection for Chronic Diseases by Telephone Inquiry (VIGITEL). CI: confidence interval, β: angular coefficient, *P*: probability value, r^2^: predictive capacity of the model

**Table 3:** Prevalence of systemic arterial hypertension in adults reporting previous medical diagnosis, stratified by age group and regions of Brazil between 2006 and 2012.

Variables	Prevalence (95% CI)							Prais–Winsten Regression			
	2006	2007	2008	2009	2010	2011	2012	β	*P*	r^2^	Trend
**Age Group (years)**											
18 to 24	4.9 (4.1; 5.7)	4.9 (3.9; 5.8)	5.0 (4.2; 5.8)	5.3 (4.2; 6.3)	5.0 (4.0; 6.1)	4.0 (3.0; 4.9)	3.8 (3.0; 4.7)	−0.18	0.134	0.81	Stable
25 to 34	9.5 (8.5; 10.6)	9.9 (8.8; 11.0)	10.4 (9.2; 11.6)	11.7 (10.2; 13.1)	8.8 (7.7; 9.9)	9.4 (8.3; 10.4)	8.8 (7.6; 9.9)	−0.17	0.390	−0.34	Stable
35 to 44	18.1 (16.7; 19.4)	19.0 (17.6; 20.4)	21.1 (19.5; 22.6)	20.9 (19.2; 22.5)	18.4 (16.9; 20.0)	19.7 (18.2; 21.2)	19.3 (17.7; 21.0)	0.09	0.731	-	Stable
45 to 54	31.9 (29.9; 33.9)	35.3 (33.3; 37.4)	37.5 (35.4; 39.5)	34.4 (32.3; 36.4)	35.3 (33.3; 37.3)	34.4 (32.9; 36.2)	34.6 (32.6; 36.6)	0.13	0.702	0.49	Stable
55 to 64	49.0 (46.3; 51.7)	50.0 (47.4; 52.5)	52.1 (49.6; 54.6)	50.8 (48.4; 53.2)	51.7 (49.3; 54.2)	50.0 (47.7; 52.2)	50.0 (47.6; 52.4)	0.10	0.689	−0.18	Stable
≥65	57.7 (55.3; 60.0)	57.2 (54.6; 59.8)	61.7 (59.3; 64.0)	63.5 (61.2; 65.8)	60.2 (57.6; 62.3)	58.7 (57.1; 61.9)	59.2 (56.9; 61.6)	0.27	0.613	−0.84	Stable
**Regions**											
North	19.7 (18.7; 20.8)	18.7 (17.6; 19.8)	18.6 (17.6; 19.7)	20.4 (19.3; 21.6)	19.2 (18.1; 20.2)	19.9 (18.8; 21.0)	18.7 (17.4; 20.0)	0.06	0.535	0.98	Stable
Northeast	22.3 (21.5; 23.2)	22.9 (22.0; 23.8)	23.5 (22.6; 24.5)	24.6 (23.7; 25.6)	23.4 (22.6; 24.3)	23.2 (22.3; 24.0)	23.9 (23.0; 24.9)	0.20	0.200	0.57	Stable
Southeast	23.8 (22.5; 25.2)	25.7 (24.3; 27.2)	28.5 (27.0; 30.0)	27.7 (26.2; 29.3)	26.3 (24.9; 27.7)	26.2 (24.8; 27.6)	25.8 (24.3; 27.3)	0.23	0.544	0.65	Stable
South	21.9 (20.5; 23.2)	22.7 (21.3; 24.0)	24.6 (23.3; 26.0)	23.6 (22.2; 25.0)	25.1 (23.8; 26.5)	24.4 (23.1; 25.7)	24.7 (23.2; 26.1)	0.43	0.014	0.97	Increase
Midwest	20.1 (18.8; 21.5)	20.8 (19.6; 21.9)	22.7 (21.4 (23.9)	24.1 (22.2; 25.9)	21.7 (19.7; 23.7)	23.2 (22.0; 24.4)	24.1 (22.7; 25.4)	0.56	0.037	0.76	Increase

**Data Source:** Surveillance of Risk Factors and Protection for Chronic Diseases by Telephone Inquiry (VIGITEL). CI: confidence interval, β: angular coefficient, *P*: probability value, r^2^: predictive capacity of the model.

**Table 4:** Prevalence of abusive consumption of alcohol by adults, stratified by age group and regions of Brazil between 2006 and 2012.

Variables	Prevalence (95% CI)							Prais–Winsten Regression			
	2006	2007	2008	2009	2010	2011	2012	β	*P*	r^2^	Trend
**Age Group (years)**											
18 to 24	18.7 (17.1; 20.3)	22.7 (20.8; 24.5)	21.3 (19.6; 23.0)	23.3 (21.4; 25.3)	22.0 (20.2; 23.8)	20.2 (18.5; 22.0)	21.8 (19.7; 23.9)	0.11	0.690	−0.84	Stable
25 to 34	21.6 (20.1; 23.1)	21.7 (20.2; 23.1)	22.1 (20.6; 23.6)	23.9 (22.3; 25.5)	24.1 (22.5; 25.7)	21.3 (19.8; 22.8)	24.7 (22.9; 26.6)	0.33	0.114	0.96	Stable
35 to 44	17.4 (16.0; 18.8)	16.7 (15.4; 17.9)	19.4 (17.9; 20.8)	20.0 (18.5; 21.5)	19.8 (18.2; 21.5)	18.2 (16.8; 19.7)	20.0 (18.3; 21.7)	0.40	0.123	0.38	Stable
45 to 54	13.2 (11.8; 14.6)	14.4 (13.0; 15.8)	15.2 (13.8; 16.7)	16.8 (15.2; 18.4)	15.9 (14.5; 17.2)	14.8 (13.5; 16.2)	16.6 (15.0; 18.1)	0.43	0.081	0.007	Stable
55 to 64	7 (5.7; 8.3)	9.4 (8.0; 10.8)	10.2 (8.6; 11.8)	10.4 (9.0; 11.8)	10.7 (9.2; 12.2)	10.6 (9.1; 12.0)	11.9 (10.3; 13.5)	0.63	0.008	0.74	Increase
≥65	2.5 (1.8; 3.2)	2.7 (1.9; 3.5)	3.3 (2.6; 4.1)	4.1 (3.2; 5.0)	4.4 (3.4; 5.3)	4.5 (3.5; 5.4)	5.5 (4.0; 5.9)	0.43	<0.001	0.94	Increase
**Regions**											
North	17.2 (16.1; 18.3)	17.3 (16.2; 18.4)	19.7 (18.4; 21.0)	18.4 (17.2; 19.6)	18.3 (17.1; 19.5)	15.9 (14.8; 17.1)	16.6 (15.4; 17.9)	−0.20	0.472	−0.24	Stable
Northeast	18.4 (17.5; 19.3)	19.2 (18.3; 20.1)	19.9 (19.0; 20.9)	20.8 (19.8; 21.8)	20.8 (19.9; 21.8)	19.2 (18.3; 20.1)	20.5 (19.4; 21.5)	0.27	0.164	-	Stable
Southeast	14.3 (13.1; 15.4)	15.3 (14.1; 16.5)	16.0 (14.7; 17.2)	17.6 (16.3; 19.0)	16.9 (15.6; 18.1)	15.9 (14.7; 17.2)	18.1 (16.6; 19.5)	0.47	0.033	0.75	Increase
South	13.3 (12.1; 14.5)	14.5 (13.2; 15.7)	12.8 (11.6; 13.9)	15.4 (14.1; 16.7)	15.3 (14.0; 16.5)	14.1 (12.9; 15.4)	15.2 (13.8; 16.6)	0.24	0.086	0.96	Stable
Midwest	15.4 (14.0; 16.7)	17.0 (15.8; 18.2)	17.3 (16.0; 18.6)	18.6 (17.0; 20.2)	18.7 (16.6; 20.8)	15.0 (13.9; 16.2)	19.3 (17.9; 20.7)	0.22	0.374	0.89	Stable

**Data Source:** Surveillance of Risk Factors and Protection for Chronic Diseases by Telephone Inquiry (VIGITEL). CI: confidence interval, β: angular coefficient, *P*: probability value, r^2^: predictive capacity of the model.

**Table 5:** Prevalence of overweight rate and obesity among adults, stratified by age group and regions of Brazil between 2006 and 2012.

**Variables**	**Prevalence of Overweight (95% CI)**							**Prais-Winsten Regression**			
	**2006**	**2007**	**2008**	**2009**	**2010**	**2011**	**2012**	**β**	***P***	**r^2^**	**Trend**
**Age group (years)**											
18 to 24	16.5 (14.8; 18.2)	16.5 (14.8; 18.2)	18.5 (16.8; 20.2)	19.4 (17.3; 21.5)	21.9 (19.7; 24.0)	19.7 (17.9; 21.6)	21.1 (19.1; 23.1)	0.85	0.006	0.90	Increase
25 to 34	28.0 (26.2; 29.7)	28.9 (27.2; 30.6)	29.9 (28.2; 31.7)	30.0 (28.2; 31.8)	31.6 (29.8; 33.4)	32.2 (30.5; 33.9)	32.3 (30.3; 34.2)	0.77	<0.001	0.99	Increase
35 to 44	36.4 (34.6; 38.3)	33.9 (32.1; 35.6)	34.7 (33.0; 36.5)	35.7 (33.8; 37.6)	36.0 (33.9; 38.0)	35.9 (34.0; 37.8)	36.0 (34.0; 37.9)	0.19	0.231	0.95	Stable
45 to 54	39.1 (36.9; 41.2)	36.4 (34.4; 38.5)	37.3 (35.3; 39.3)	37.4 (35.3; 39.5)	36.7 (34.6; 38.8)	36.9 (34.9; 38.8)	38.2 (36.2; 40.3)	−0.06	0.669	0.99	Stable
55 to 64	39.7 (36.8; 42.5)	38.4 (35.8; 41.1)	38.3 (35.8; 40.9)	38.5 (36.0; 41.0)	41.5 (38.9; 44.1)	39.6 (37.2; 41.9)	37.0 (34.7; 39.3)	−0.09	0.768	0.06	Stable
≥65	36.6 (34.0; 39.2)	37.1 (34.3; 39.9)	36.5 (33.9; 39.1)	38.2 (35.6; 40.9)	38.7 (36.2; 41.3)	37.5 (35.1; 40.0)	39.0 (36.6; 41.5)	0.35	0.011	0.99	Increase
**Regions**											
North	29.1 (27.8; 30.4)	29.8 (28.5; 31.2)	31.4 (29.9; 32.8)	31.7 (30.2; 33.1)	32.4 (31.0; 33.7)	33.0 (31.6; 34.4)	33.0 (31.4; 34.6)	0.68	0.001	0.83	Increase
Northeast	28.9 (27.9; 30.0)	29.8 (28.8; 30.9)	30.3 (29.3; 31.4)	31.2 (30.1; 32.3)	31.9 (30.9; 33.0)	32.4 (31.4; 33.4)	33.2 (32.1; 34.3)	0.70	<0.001	0.99	Increase
Southeast	32.9 (31.2; 34.5)	31.1 (29.5; 32.7)	32.2 (30.6; 33.8)	33.1 (31.4; 34.7)	34.5 (32.8; 36.2)	32.8 (31.2; 34.4)	33.4 (31.6; 35.1)	0.28	0.128	0.93	Stable
South	33.1 (31.5; 34.7)	31.4 (29.8; 33.0)	32.5 (30.9; 34.1)	32.9 (31.2; 34.5)	33.5 (31.9; 35.2)	34.7 (33.1; 36.2)	35.2 (33.4; 37.0)	0.52	0.009	0.96	Increase
Midwest	30.0 (28.2; 31.8)	29.5 (28.0; 31.0)	30.7 (29.3; 32.2)	31.5 (29.2; 33.2)	33.0 (30.6; 35.5)	34.0 (32.5; 35.5)	33.4 (31.9; 35.0)	0.82	<0.001	0.99	Increase
** Variables **	** Prevalence of Obesity (95% CI) **							** Prais-Winsten Regression **			
	** 2006 **	** 2007 **	** 2008 **	** 2009 **	** 2010 **	** 2011 **	** 2012 **	** β **	*** P ***	** r^2^**	** Trend **
** Age group (years) **											
18 to 24	4.2 (3.4; 4.9)	4.0 (3.2; 4.7)	4.4 (3.6; 5.2)	6.2 (5.1; 7.3)	5.1 (4.1; 6.0)	5.7 (4.7; 6.7)	7.2 (5.9; 8.6)	0.46	0.003	0.93	Increase
25 to 34	10.0 (8.7; 11.2)	10.9 (9.7; 12.1)	11.1 (9.8; 12.4)	11.3 (10.0; 12.6)	12.3 (11.1; 13.4)	13.5 (12.2; 14.8)	14.8 (13.2; 16.3)	0.77	0.001	0.89	Increase
35 to 44	12.4 (11.3; 13.6)	14.7 (13.2; 16.2)	14.9 (13.5; 16.3)	15.4 (14.0; 16.9)	16.2 (14.8; 17.7)	18.9 (17.4; 20.5)	19.7 (18.0; 21.4)	1.13	<0.001	0.93	Increase
45 to 54	17.6 (14.3; 17.7)	19.1 (17.3; 20.8)	18.3 (16.6; 20.0)	17.3 (15.7; 18.9)	21.4 (19.5; 23.2)	21.1 (19.5; 22.7)	22.3 (20.6; 24.1)	0.89	0.004	0.96	Increase
55 to 64	14 (15.2; 19.6)	19.5 (18.5; 21.5)	20.9 (18.7; 23.1)	21.2 (19.0; 23.4)	20.1 (18.2; 22.0)	20.5 (18.7; 22.4)	23.3 (21.2; 25.4)	0.70	0.027	0.36	Increase
≥65	13.6 (14.0; 18.1)	13.3 (12.5; 15.1)	17.4 (15.3; 19.5)	17 (15.0; 19.1)	19.4 (17.2; 21.6)	17.6 (15.8; 19.5)	18.3 (16.5; 20.0)	0.71	0.013	0.94	Increase
** Regions **											
North	12.9 (11.9; 13.9)	13.3 (12.3; 14.2)	14.0 (13.0; 15.1)	14.4 (13.3; 15.4)	16.3 (15.2; 17.4)	16.7 (15.6; 17.8)	17.9 (16.6; 19.2)	0.86	<0.001	0.98	Increase
Northeast	11.4 (10.7; 12.1)	12.3 (11.5; 13.1)	13.2 (12.4; 14.0)	13.8 (13.0; 14.6)	14.7 (13.9; 15.5)	15.5 (14.7; 16.3)	16.7 (15.8; 17.6)	0.84	<0.001	0.99	Increase
Southeast	11.5 (10.4; 12.7)	13.2 (12.0; 14.4)	13.6 (12.3; 14.8)	14.4 (13.2; 15.7)	15.0 (13.8; 16.2)	15.8 (14.5; 17.0)	17.7 (16.3; 19.1)	0.85	<0.001	0.99	Increase
South	12.4 (11.3; 13.5)	12.8 (11.7; 14.0)	14.2 (13.0; 15.3)	13.4 (12.3; 14.6)	15.9 (14.7; 17.1)	16.5 (15.3; 17.7)	16.9 (15.5; 18.2)	0.83	<0.001	0.99	Increase
Midwest	10.9 (9.8; 12.0)	11.7 (10.8; 12.7)	12.5 (11.5; 13.4)	11.5 (10.3; 12.7)	13.0 (11.6; 14.3)	15.0 (13.9; 16.1)	15.6 (14.5; 16.7)	0.76	0.006	0.73	Increase

**Data Source:** Surveillance of Risk Factors and Protection for Chronic Diseases by Telephone Inquiry (VIGITEL). CI: confidence interval, β: angular coefficient, *P*: probability value, r^2^: predictive capacity of the model.

## Data Availability

All data were taken from public systems of the Federal Government of Brazil, through the Information Technology Department of the Brazilian Unified Health System (DATASUS), which can be accessed through the link: https://datasus.saude.gov.br/ (accessed on 31 May 2018).
